# Fourier Transform Infrared Spectroscopy as a Tool to Study Milk Composition Changes in Dairy Cows Attributed to Housing Modifications to Improve Animal Welfare

**DOI:** 10.3390/foods10020450

**Published:** 2021-02-18

**Authors:** Mazen Bahadi, Ashraf A. Ismail, Elsa Vasseur

**Affiliations:** 1 McGill IR Group, McGill University, Sainte Anne de Bellevue, QC H9X 3V9, Canada; ashraf.ismail@mcgill.ca; 2 Department of Animal Science, McGill University, Sainte Anne de Bellevue, QC H9X 3V9, Canada; elsa.vasseur@mcgill.ca

**Keywords:** milk FTIR spectroscopy, animal welfare, biomarker

## Abstract

Animal welfare status is assessed today through visual evaluations requiring an on-farm visit. A convenient alternative would be to detect cow welfare status directly in milk samples, already routinely collected for milk recording. The objective of this study was to propose a novel approach to demonstrate that Fourier transform infrared (FTIR) spectroscopy can detect changes in milk composition related to cows subjected to movement restriction at the tie stall with four tie-rail configurations varying in height and position (TR1, TR2, TR3 and TR4). Milk mid-infrared spectra were collected on weekly basis. Long-term average spectra were calculated for each cow using spectra collected in weeks 8–10 of treatment. Principal component analysis was applied to spectral averages and the scores of principal components (PCs) were tested for treatment effect by mixed modelling. PC7 revealed a significant treatment effect (*p* = 0.01), particularly for TR3 (configuration with restricted movement) vs. TR1 (recommended configuration) (*p* = 0.03). The loading spectrum of PC7 revealed high loadings at wavenumbers that could be assigned to biomarkers related to negative energy balance, such as β-hydroxybutyrate, citrate and acetone. This observation suggests that TR3 might have been restrictive for cows to access feed. Milk FTIR spectroscopy showed promising results in detecting welfare status and housing conditions in dairy cows.

## 1. Introduction

Animal welfare status is typically assessed using visual observations such as body injuries scores requiring an on-farm visit by a trained assessor. Alternatively, milk samples are already collected routinely by dairy herd improvement agencies to check quality and safety. It would be convenient to be able to predict cow welfare status directly in milk samples [1]. The specific objective of this paper was to develop a methodology to detect cow welfare status in milk composition by the analysis of milk Fourier transform infrared (FTIR) spectra to detect the effects of dairy cows housing conditions with a varying level of welfare. We chose the tie-rail configuration as an example of a variable housing element to develop this methodology. The tie-rail controls the forward location of a cow in her stall and facilitates or obstructs the cow movement and access to feed. We hypothesized that cows more restricted in their movement, and therefore with a reduced level of welfare, might lead to detectable changes in milk composition. We also hypothesized that if the restriction of movement led to reduced feed access, changes in milk composition will be most likely detectable, since reduced carbohydrate intake results in reduced plasma glucose concentration, which might result in decreased lactose concentration in milk [2]. Additionally, a reduction in plasma glucose concentration might elevate body fat mobilization, which is characterized by increased non-esterified fatty acids (NEFA), β-hydroxybutyrate (BHB) and reduced glucose concentration in the plasma, as well as decreased protein and increased acetone, BHB, C18:1 and C16:0 in milk [2,3]. Monitoring changes of milk composition has already been proposed as a tool to monitor important health issues in dairy cows, such as negative energy balance, ketosis and mastitis [4], and changes to the fatty acids profile of milk fat have been reported as a response to changes in the type of forage (i.e., odd, branched and trans fatty acids) and the energy status of cows during lactation (i.e., saturated fatty acids vs. monounsaturated fatty acids) [5]. To test our hypothesis, a methodology was needed to detect trends in changing milk composition, as early as possible, that might result from changes in the welfare status due to modifications in the housing conditions of dairy cows, in our case, the tie-rail configuration. The ultimate goal of this study was to develop precision markers that could be routinely recorded to detect cows experiencing a good or poor level of welfare, which will enable dairy herd improvement agencies to offer new services to dairy producers to improve animal welfare based on routinely collected milk FTIR spectra. According to our knowledge, no such application of milk FTIR spectroscopy has been reported in the literature at the time of study.

Over the past six decades, multiple applications based on milk analysis by infrared (IR) spectroscopy have been developed. Quantitative milk analysis by mid-infrared (MIR) spectroscopy was proposed in 1960s as a rapid and cheap method [6,7], which did not involve hazardous chemicals or sample preparation. At that time, milk fat, protein, lactose, and solids-not-fat were determined by IR spectroscopy. In the 1990s, Fourier transform infrared (FTIR) spectroscopy was proposed for milk analysis in combination with partial least squares (PLS) regression [8], which later became the basis of the official method for milk analysis by IR spectroscopy [9,10]. Over the years, many studies attempted to predict milk components other than the major ones (i.e., fat, protein, lactose) from milk FTIR spectra, such as individual and groups of fatty acids [11,12,13,14,15,16,17], fatty acid chain length and unsaturation [18,19], milk protein composition and their genetic variants [20], milk lactoferrin as an indicator of mastitis [21], major mineral contents of milk (i.e., Ca, K, Mg, Na and P) [22,23] and milk acetone, BHB and citrate, as biomarkers for a cow’s metabolic state [24]. Other studies focused on predicting technological properties from milk FTIR spectra, such as fat globule particle size in homogenized milk [25], milk coagulation properties [23,26,27,28,29], titratable acidity and pH in milk [26]. All these studies had two common aspects: (1) they all relied on PLS to predict the trait or property of interest from milk FTIR spectra; and (2) they all targeted concentrations of milk components or the technological properties affected by physical attributes of milk components (e.g., fat globules and protein micelles sizes) that led to changes in milk FTIR spectra (e.g., IR band absorbance intensity or band shifts), which could be correlated to the trait or property of interest. More recently, researchers started to investigate the potential of algorithms other than PLS to predict milk components and technological properties from milk FTIR spectra. Ferragina et al. (2015) demonstrated that Bayesian regression models outperformed PLS in predicting milk components and technological properties [30]. Soyeurt et al. (2020) compared the performance of a PLS model to those built by combining PLS factors with linear and polynomial support vector machines and artificial neural networks to predict milk lactoferrin content [31]. 

Other studies focused on predicting important indicators for the dairy farming sector that are indirectly correlated to changes in milk FTIR spectra, such as methane production in the rumen [32,33], body energy status [34], feed efficiency [35], dry matter intake and feed residual [36], and blood metabolites (e.g., BHB, urea, and NEFA) in early-lactation cows [37]. In these studies, the PLS algorithm was used to predict the indicator of interest. Other studies implemented clustering (e.g., K-means clustering) and classification (partial least squares discriminant analysis and random forests) algorithms to predict dairy farming indicators from milk FTIR spectra. Such applications included the classification of milk according to its production system (i.e., fresh grass feeding, pasture grazing and organic farming) [38], the prediction of the metabolic status and energy balance of dairy cows [39], metabolic profiling [40] and clustering [41] of early-lactation dairy cows, prediction of physiological imbalance in Holstein dairy cows [42], the prediction of cows’ pregnancy status [43], fertility [44] and the likelihood of conception to first insemination [45]. Other studies looked into the correlation between milk FTIR spectra and lameness in later lactation [46], milk MIR predicted gastro-enteric methane production and the technical and financial performance of commercial dairy herds [47], diagnosing pregnancy status in dairy cows [48], and predicting the heritability and genetic correlation of milk coagulation properties from milk FTIR spectra [49]. 

In our study, we propose a novel approach for the implementation of milk FTIR spectroscopy as a tool to monitor dairy cows’ welfare status. A milk FTIR spectrum contains information about all milk molecules that contain covalent bonds that absorb IR energy; hence, this FTIR spectrum will provide insights about the chemical composition of a milk sample beyond the concentrations of a limited number of milk components. Our hypothesis was that milk FTIR spectra can capture changes in milk composition that can be attributed to the modifications of stall housing conditions and changing welfare. We propose combining principal component analysis (PCA) as a multivariate analysis tool with mixed modelling to identify and associate the relevant spectral features from milk spectra to different housing conditions in tie-stalls as a proxy for changing welfare status. Specific aspects related to animal behavior and welfare outcomes are discussed in an accompanying publication [50]. 

## 2. Materials and Methods 

### 2.1. Experimental Setup

The animal trial was conducted at the Macdonald Campus Dairy Complex of McGill University (Sainte-Anne-de-Bellevue, QC, Canada). Details on the experimental handling were described elsewhere [50]. Briefly, 48 lactating Holstein cows were assigned to 4 tie-rail configurations varying in height and position. Tie-rail configurations were TR1 (122 and 36 cm; height from the stall base and forward position from the manger well, respectively), TR2 (122 and 18 cm), TR3 (112 and 18 cm), and TR4 (112 and 36 cm). While tie-rail configuration TR1 was based on the current recommendation for tie-stalls in Canada as outlined in the Dairy Code of Practice [51], TR2 reflected the tie-rail position most found on Quebec dairy farms [52], and TR3 and TR4 were designed to increase the opportunity of movements of the cow in the stall and hence, improve cow comfort and welfare relative to treatments TR1 and TR2, respectively. Cows were assigned to 6 blocks to account for parity (primiparous: n = 12, multiparous: n = 36), days in milk (DIM; early: 0–100 d, mid: 101–200 d or late: 201–305 d), and location in the barn prior to the start of the experiment. Cows were in trial for 10 weeks with 24 cows starting in summer 2016 (period 1: 25 July–3 October) and the remaining 24 cows starting in fall 2016 (period 2: 10 October–19 December). 

### 2.2. Housing and Management

Cows were housed in two separate rows of tie-stalls facing the barn wall equipped with rubber mats with wood shavings supplemented once a day. Stall dimensions were adapted to the cow size following current recommendations outlined in the Dairy Code of Practice [51]. Cows were fed a total mixed ration four times daily with feed pushed up 6 times per day to ensure food availability. Water was accessible ad libitum from a self-filling water bowl. Cows were milked in-stall twice daily at a 12 h interval. Detailed housing and management specifications can be found in an accompanying publication [50]. 

### 2.3. Milk Analysis

One composite milk sample per week was collected from each cow participating in the trial. The sample consisted of milk collected during the evening milking and the morning milking of the next day. The collected milk samples were analyzed for milk composition by FTIR spectroscopy at Lactanet laboratory (Sainte-Anne-de-Bellevue, QC, Canada) using the same CombiFoss FT+ analyzer (FOSS, Hillerød, Denmark). The concentrations of fat (g/100 g milk), protein (g/100 g milk), lactose (g/100 g milk), urea (mg/dL milk) and BHB (mmol/L) were determined.

### 2.4. Milk FTIR Spectra, Outliers Check and Spectral Pre-Treatments

The full FTIR spectra for the respective milk samples were collected and each FTIR spectrum contained of 1060 spectral variables between 5008 and 925 cm^−1^. The spectra were visualized using the OMNIC^TM^ software (version 7.3; Thermo Fisher Scientific, Waltham, MA, USA) and PCA in JMP Pro 13.2.1 (SAS Institute, Cary, NC, USA) was used as a tool to detect spectral outliers. In the PCA scores plot (PC1 vs. PC2), all samples clustered randomly at the origin of the PCA space, which was an indicator of the absence of spectral outliers. 

Only spectral regions containing information related to milk composition were retained for spectral analysis, which were 3061–2803 cm^−1^; 1797–1681 cm^−1^; and 1612–925 cm^−1^ [12,15,23,26,31,32,38]. The total number of spectral variables that were retained for analysis was 278 wavenumbers. Codes were written in MATLAB R2018a (MathWorks, Natick, MA, USA) to apply, separately or combined, the differential first derivative with a derivative window of 1 and vector normalization [53] as pre-treatments to milk spectra. As a result, four sets of milk FTIR spectra were obtained: raw, vector normalized raw (VN), first derivative (FD), vector normalized first derivative (VN-FD). 

In addition, short-term and long-term average spectra were calculated for each cow. The short-term and long-term averages included the spectra of samples collected from week 1 to week 3 and from week 8 to week 10, respectively. These averages were calculated for raw, VN raw, FD and VN-FD spectral sets. 

### 2.5. Statistical Model

Data of this trial were analysed under the following statistical model:

(1) $$Y_{ijk}=\mu +trt_{i}+start_{j}+block_{kji}+e_{ijk}$$

where $trt_{i}$ was the fixed effect of the $i^{th}$ TR configuration treatment, $start_{j}$ was the fixed effect of the $j^{th}$ start date, $block_{kji}$ was the fixed effect of $k^{th}$ parity, DIM and location in the barn from the $j^{th}$ start date (i.e., period 1 or 2) on the $i^{th}$ TR configuration treatment and $e_{ijk}$ was the random residual error [50]. The significance level was defined at *p* ≤ 0.05.

Originally, the statistical model of the trial included the fixed effect of the week and the random effect of the cow [50]. However, due to the ongoing changing milk composition during the lactation from one week to another, it was decided to calculate an average spectrum for each cow for the first and last three weeks of each start date of the trial to evaluate the short- (week 1–3), and long-term effects (week 8–10) of the stall configuration treatments. This approach reduced the sources of variability in milk composition related to changes in lactation stage as the trial was progressing. 

### 2.6. Combined Mixed Model and PCA for Spectral Analysis

**Figure 1:**
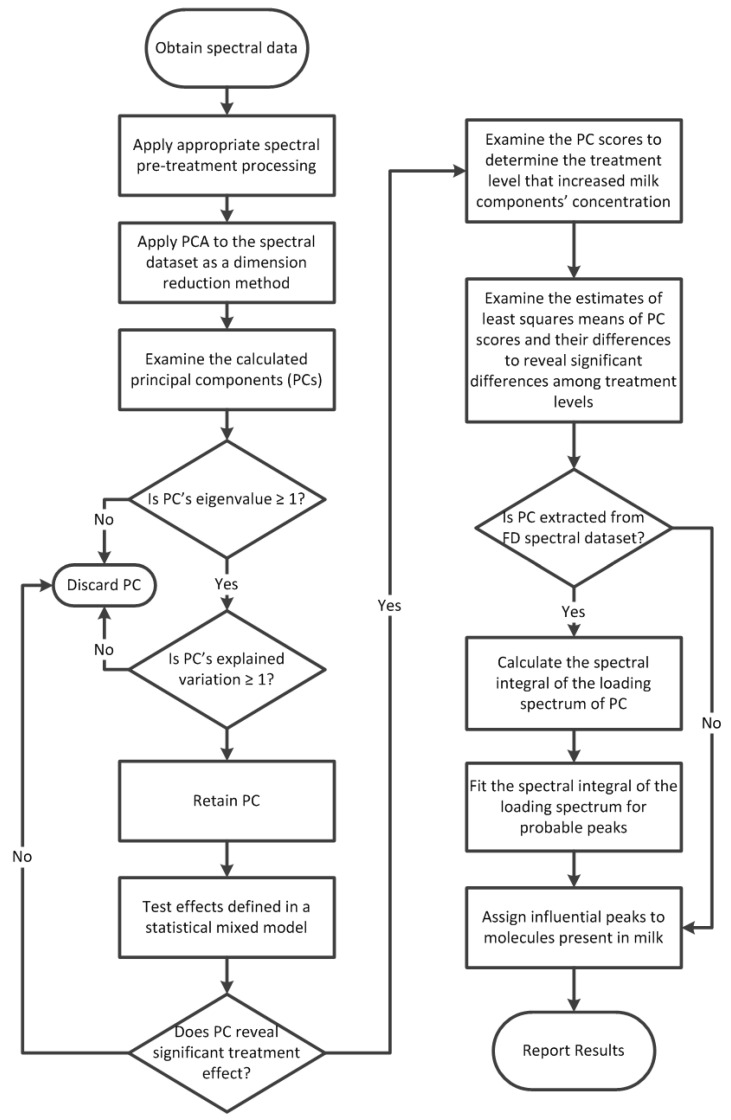
Workflow of the hybrid data analysis approach of the FTIR milk spectral data to detect a treatment effect. PCA = principal component analysis, PC = principal component, FD = first derivative.

A novel approach was developed to detect the TR configuration treatment effect on milk FTIR spectra by combining multivariate analysis with mixed modelling (Figure 1). In JMP Pro 13.2.1., PCA was applied to the four versions (i.e., raw, FD, VN raw, VN-FD) of the short- and long-term spectral averages as a dimension reduction method to reduce the number of spectral features that would be tested by the mixed model for the treatment effect [54]. Scores of the principle components (PCs) were calculated through the decomposition of the original spectral matrix *X* (i.e., milk FTIR spectra) that contains i samples and j variables as follows [55]:

(2) $$X=f_{1}q_{1}^{T}+f_{2}q_{2}^{T}+f_{3}q_{3}^{T}+t_{l}p_{l}^{T}$$

where l≤min {i,j}, l is the mathematical rank of the spectral matrix *X*, $f_{1}q_{1}^{T}$ is PC1 that describes the greatest amount of variance in *X*, $f_{2}q_{2}^{T}$ is PC2 that describes the second largest amount of variance in *X* and so on. $f_{l}$ are the sample scores in the space defined by the *l^th^* PC and $q_{l}$ are the loadings of the original variables (i.e., spectral variables or wavenumbers in *X*) for the lth PC. 

When PCs containing meaningful information are retained and noisy PCs are discarded, the general description of the PCA model becomes as follows:

(3) $$X=f_{1}q_{1}^{T}+f_{2}q_{2}^{T}+\cdots +f_{K}q_{K}^{T}+E=F_{K}Q_{K}^{T}+E$$

where *K* is the number of the retained meaningful PCs and *E* is the residual matrix that contains the noisy PCs. From Equation (3), we can say that each PC consists of a vector of scores (*f*) and a vector of loadings (*q*). Both vectors contain information that provided answers to questions related to the investigated problem. PCs with an eigenvalue equal or greater than 1 and that explained at least 1% of the variance in the spectral dataset were saved for testing by the mixed model. Thus, only PCs that contained meaningful information from milk FTIR spectra were retained for statistical analyses and PCs that mainly contained noise were discarded. The mixed procedure in SAS 9.4 (SAS Institute, Cary, NC, USA) was utilized to test the retained PC scores for the treatment effect. If a PC had revealed a significant treatment effect at *p* ≤ 0.05, then the least squares means of its scores were examined to determine the treatment levels that were significantly different from the other levels using a Scheffé adjustment for multiple comparisons. The influential spectral features could be directly extracted from the loading spectrum of the PC that had revealed the significant treatment effect if it was obtained from a raw spectral dataset. If this PC was obtained from an FD spectral dataset, then the spectral integral of the PC’s loading spectrum had to be calculated before extracting the influential spectral features. The cumulative trapezoidal numerical integration function in MATLAB R2018a (MathWorks, Natick, MA, USA) was used to calculate the spectral integral for the loading spectrum in question. If the integrated loading spectrum had produced wide humps with no clear peaks, the Peak Resolve feature in OMNIC^TM^ 7.3 (Thermo Fisher Scientific, Waltham, MA, USA) was used to fit the integrated loading spectrum for probable peaks. To do so, the Voigt function [56] with low or high sensitivity, was used and the baseline was set to none. The noise and the full width at half height of the narrowest peak in the region of interest were determined by the software. The fitting process was repeated several times until an acceptable residual spectrum was obtained.

### 2.7. Interpretation of Spectral Features in PCA Loading Spectra

Bulk tank raw milk was obtained from the Dairy Research Complex, Macdonald Campus, McGill University (Ste.-Anne-de-Bellevue, QC, Canada). Milk samples of 35 mL were spiked with different amounts of minor milk components and aqueous solutions with different concentrations of these chemicals were prepared. Their FTIR spectra were recorded at Lactanet laboratory (Ste.-Anne-de-Bellevue, QC, Canada) by the same milk analyzer that was used for the trial’s milk samples. Linoleic acid was chosen as an example of unsaturated fatty acid. It must be noted that different fatty acids do not produce distinct FTIR signals from each other, especially when they are present in a mixture of fatty acids [15]. Urea, ammonium, creatine, histamine, orotic acid and hippuric acid were chosen as representatives of nonprotein nitrogen (NPN) compounds present in milk [57]. Milk BHB, acetone, citrate and acetate were chosen as markers for energy intake-related issues in dairy cows [24]. In addition, phosphate, lactose, glucose, and galactose were also chosen. OMNIC^TM^ 7.3 (Thermo Fisher Scientific, Waltham, MA, USA) was used to calculate variance spectra and the second derivative for the collected spectra, and the Find Peaks functionality was used to determine IR band centers whose intensity was increasing as a function of the increased concentration of the compound in milk and in the aqueous solution.

### 2.8. Ethics Statement

Use of animals and all procedures were approved by the Animal Care Committee of McGill University and affiliated hospitals and research institutes (protocol #2016–7794). All aspects of this study met the standards established by the Canadian Council on Animal Care to ensure the humane and ethical use of animals in research.

## 3. Results

### 3.1. Interpretation of Spectral Features in Loading Spectra

Table 1 summarizes the spectral features of minor milk components observed in the second derivative of FTIR spectra of milk samples spiked with these molecules and their respective aqueous solutions. These observed features were used to identify the influential features and their respective molecules in the loading spectra of principal components that revealed a significant treatment effect in this study.

### 3.2. Spectral Analysis 

Table 2 summarizes the PCs that were extracted from the raw, FD, VN raw and VN-FD spectral datasets of the short-term and long-term milk samples spectral averages of the tie-rail trial. The PCA yielded five, seven, four and nine PCs from the raw, FD, VN raw and VN-FD long-term spectral average datasets that explained 97.35, 94.92, 96.99 and 95.44% of the variance in the spectral datasets, respectively. These PCs, whose eigenvalue and percentage of explained variance ≥1, represent the sources of systemic variation in their respective spectral datasets that were separated from noise and that were tested for the treatment, start (i.e., period 1 or 2) and block effects by the SAS Mixed procedure. PC6 (*p* = 0.0371), PC4 (*p* = 0.0462) and PC7 (*p* = 0.0106) extracted from long-term FD, VN raw, VN-FD spectral average datasets, respectively, revealed significant treatment effect. Among the three PCs that revealed a significant treatment effect, PC7 (*p* = 0.0106) isolated from the long-term VN-FD spectral dataset revealed the strongest treatment effect, and unlike the other two PCs, the start (*p* = 0.5590) and block (*p* = 0.0600) had insignificant effects on it. This observation can be explained by the fact that FD exposed more details in the spectral dataset and VN eliminated variability not related to the chemical composition of milk samples, which facilitated the isolation of the treatment effect from the other two studied effects on the FTIR spectra of milk. For these reasons, PC7, isolated from the long-term VN-FD spectral dataset, was considered for further analysis to determine the treatment levels that were significantly different from each other and the spectral features that were responsible for these differences. PC7 explained 1.37% of the variance in its respective dataset, which suggests that the treatment effect is limited, but the change in milk composition that can be attributed to this effect was captured by milk FTIR spectra at an early point in time before these changes in milk composition may reach critical thresholds.

Table 3 and Table 4 summarize the least squares means and their differences produced by the mixed procedure for the scores of PC7 extracted from the long-term VN-FD spectral average dataset that revealed a significant treatment effect. These tables show that the PC7 scores of milk samples of cows enrolled in TR3 were significantly different from the scores of milk samples of other treatments (*p* = 0.0038) and the Scheffé adjusted *p* value shows that TR3 is significantly different from TR1 (*p* = 0.0332).

**Figure 2:**
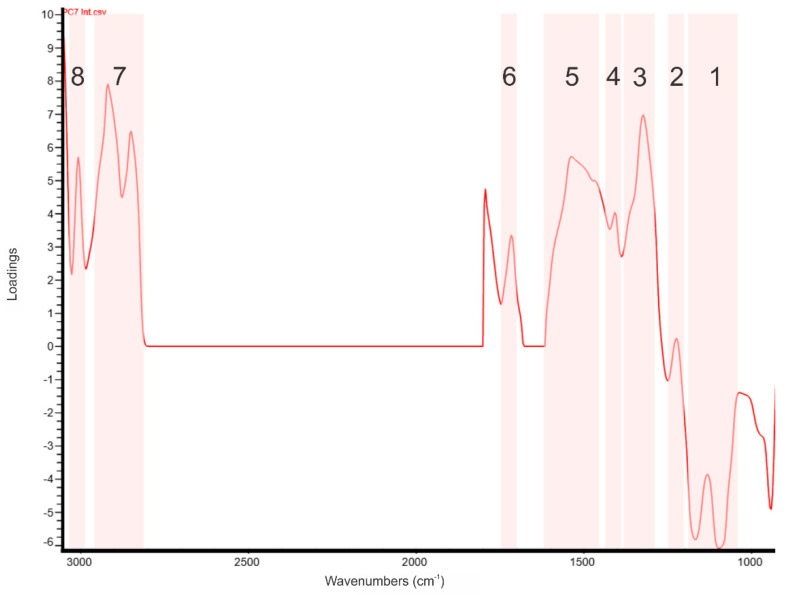
The spectral integral of the PC7 loading spectrum extracted from the long-term VN-FD spectral average dataset for the tie-rail trial. Shaded regions can be assigned to (1) lactose 1200–1000 cm^−1^, (2) acetone ~1237 cm^−1^, (3) citrate, BHB and acetone 1390–1250 cm^−1^, (4) BHB ~1404 cm^−1^, (5) fatty acids and carboxylate ion in citrate, BHB, free fatty acids and acetate 1618–1424 cm^−1^, (6) carboxylic group of free fatty acids ~1716 cm^−1^, (7) CH stretching of fatty acids 3000–2800 cm^−1^, and (8) =C–H stretching of fatty acids ~3008 cm^−1^.

Inspection of the integral of PC7 loading spectrum (Figure 2) revealed high positive loadings at the following wavenumbers: 3008, 2919, 2851, 1716, and 1407 cm^−1^, which can be theoretically assigned to the following IR bands: the C–H stretching in the alkene (olefinic) bond (C=C–H) in unsaturated fatty acids, the asymmetrical stretching ($\nu_{as}CH_{2}$) of the methylene group in fatty acids, symmetrical stretching ($\nu_{s}CH_{2}$) of the methylene group in fatty acids, the C=O stretching vibration in the carboxyl functional group in free fatty acids and the symmetrical stretching of the carboxylate ion or the C–O–H bending in BHB, respectively [58]. Experimentally, these assignments can be confirmed by the results of the spiking experiment reported in Table 1. For example, the aqueous solutions of BHB and spiked milk samples with BHB revealed IR bands with increased intensity centered at 1404 and 1405 cm^−1^, respectively. These wavenumbers are within band 4 of the increased loadings in Figure 2. 

The peak fitting process was applied to regions 1618–1424, 1390–1250 and 1250–1180 cm^−1^, which did not show any clear peaks. In the first region, a probable peak was detected at 1237 cm^−1^, which can be assigned to the stretching of the C–C–C group and the bending of C–C(=O)–C in the C–C–C group in acetone [58]. In the second region, peaks were detected at 1287, 1317 and 1372 cm^−1^, which can be assigned to the C–O stretching that appears in the FTIR spectrum of the aqueous solution of citrate, the C–O stretching in BHB and the symmetrical ($\delta_{s}CH_{3}$) bending vibration of the methyl group in acetone, respectively [58]. In the third region, peaks were detected at 1460 and 1541 cm^−1^. The 1460 cm^−1^ peak can be assigned to the asymmetrical bending vibration of the methyl group ($\delta_{as}CH_{3}$) or to the scissoring band of the methylene group ($\delta_{s}CH_{2}$) in fatty acids [58]. On the other hand, the 1541 cm^−1^ peak can be assigned to the symmetrical stretching of the carboxylate ion that was found in citrate, BHB, free fatty acids and acetate [58]. In addition, negative loadings were observed in the region between ~1040 and ~1100 cm^−1^, which is dominated by lactose absorption in milk FTIR spectra [59].

To summarize, the results of the spectral analysis suggests that the average FTIR spectra of milk samples collected from cows enrolled in TR3 in the last three weeks of the trial (long-term effect) had significance differences compared to the average FTIR spectra of milk samples collected from cows enrolled in TR1 during the same period. The loading spectrum of the principal component that revealed the significant treatment effect showed high positive loadings for spectral features that can be assigned to fatty acids, BHB, acetone and citrate. It also showed negative loadings for spectral features that can be assigned to lactose. This observation suggests that lactose had an inversed relationship with fatty acids, BHB, acetone and citrate in milk samples collected during the last three weeks of the trial (i.e., long-term effect of the tie-rail configuration treatment application). In addition, treatment TR3 tended to differ from treatment TR2 (*p* = 0.0711, less restricted than TR3 in its height from the stall base) and treatment TR4 (*p* = 0.0622, less restricted than TR3 in its forward position from the manger wall).

## 4. Discussion

Combining PCA and mixed modelling proved to be a successful strategy to assess the relationship between housing conditions and the trends of changes in milk composition in dairy cows. The spectral analysis methodology described in Figure 1 could address two points required for this specific application. First, the analysis was directly applied to the FTIR spectral data of milk, which contain more information related to milk composition than the concentrations of specific milk components reported by milk analyzers. The FTIR spectrum of a milk sample is a chemical fingerprint of that sample; hence, it can provide information about milk components other than the major ones (e.g., fat, protein, and lactose) and some minor ones (e.g., urea and BHB) that are routinely analyzed by IR milk analyzers. Second, this methodology enabled hypothesis testing and assessing multiple effects on spectral data through mixed modelling while retaining the multivariate structure of the spectral data. To our knowledge, at the time of the trial, no tool was available to test random effects on spectral data and provide statistical significance for the tested factors. 

In the following section, we will discuss how PCA principal components, loadings, and scores were used in combination with mixed modelling to answer specific questions related to the problem under investigation and the interpretation of the results of this methodology of spectral analysis. 

### 4.1. Effect of Tie-Rail Configuration on Milk FTIR Spectral Data

PCA was applied to the four versions of the milk spectral dataset (i.e., raw, FD, VN raw, VN-FD) to reduce their dimensionality. PCA created a new dataset of orthogonal variables called principal components (PCs), which were obtained as linear combinations of the original variables. Those PCs described the same variance structure that was described by 278 inter-correlated dependent spectral variables, which were present in the original spectral dataset [55]. Each of these PCs described a unique source of variation that affected the composition of milk samples and their FTIR spectra. However, these sources of variation might have been meaningful factors that led to systemic variation in milk composition or they might have been noise related to the experimental procedure or the FTIR analyzer. A PC was considered describing meaningful information or a systemic source of variation when it explained 1% or more of the variance in the dataset and when its eigenvalue was equal to or greater than 1. These criteria were recommended by SAS statistical software developers [54,60,61], among others [55]. As a result, PCA dimensionality reduction separated meaningful information from noise in milk FTIR spectra and a maximum of 10 PCs were retained. This reduction in variable numbers (i.e., from 278 to a maximum of 10) facilitated testing milk FTIR spectra for fixed and random effects by the Mixed procedure. 

PCA scores are the projections of samples onto PCs and they can be considered as values of the new variables (i.e., PCs) for the samples. Samples with scores close to each other indicate similar attributes described by that PC. Greater differences in scores indicate more differences in these attributes. Typically, scores are visualized in two- or three-dimensional scatter plots where each axis represents a PC and where theses plots display the pattern of similarity of samples as points in a map [55]. In the methodology described in Figure 1, the scores of each PC were used as input for the mixed procedure, since scores can be used as input for other algorithms [54,60,61]. The output of the mixed procedure revealed that the tie-rail configuration treatment had a significant effect on the scores of PC6 (*p* = 0.0371), PC4 (*p* = 0.0462) and PC7 (*p* = 0.0106) extracted from long-term FD, VN raw, VN-FD spectral average datasets, respectively. The estimates of the least squares means and their differences calculated by the mixed procedure revealed that PC7 scores of milk samples collected from cows enrolled in TR3 were significantly different from the scores of milk samples enrolled in other treatment levels and specially those enrolled in TR1. This observation indicates that there are significant differences in the attributes of milk samples belonging to TR1 and TR3 that are affected by the systemic source of variation described by PC7. We further observed a difference, albeit less clear, between TR3 and treatments TR2 (*p* = 0.0711) and TR4 (*p* = 0.0622). It should be noted that movement was restricted for either of these treatments but TR2 was less restricted than TR3 in its height from the stall base and TR4 was less restricted than TR3 in its forward position from the manger wall.

### 4.2. Spectral Features Associated to Differences in Milk FTIR Spectra Attributed to Changes in Tie-Rail Configuration

PCA loadings represent the weights that linearly combine the spectral variables to calculate the scores for every sample for a specific PC, in other words, they represent the contribution of each spectral variable to the sample scores of a specific PC. Hence, loadings can provide insights about the spectral variables capturing differences in the attributes of samples that are greatly affected by the systemic source of variation described by that specific PC. The inspection of the loading spectrum of the PC that had revealed a significant treatment effect helped to identify the actual spectral features that captured differences in milk FTIR spectra attributed to changes in tie-rail configuration. In this trial, the integral of the PC7 loading spectrum revealed high positive loadings at 3008, 2919, 2851, 1716, and 1407 cm^−1^ and negative loadings between ~1040 and ~1100 cm^−1^. This observation suggests that differences in milk sample attributes affected by the tie-rail configuration treatment were captured by those spectral variables.

### 4.3. Assigning Potential Milk Components to Wavenumbers with High PC Loadings

The final aim was to identify milk molecules with covalent bonds that absorb IR energy at wavenumbers showing high loadings in the PC previously attributed to change in tie-rail configuration. This was performed by extracting the spectral features of milk components observed in the second derivative of FTIR spectra of milk samples spiked with increased concentrations of these molecules and their respective aqueous solutions. These observed features were used to assign potential milk components to the wavenumbers that had high loadings in the PC that revealed a significant treatment effect. It must be noted that these wavenumbers need not be an exact match for the IR band centers of the spectral features reported in Table 1. If a wavenumber with high loadings falls within the FTIR band of a specific bond of a molecule then they could be assigned to that molecule. In this trial, the loadings of PC7 suggested that lactose had an inversed relationship with fatty acids, BHB, acetone and citrate in milk samples collected from cows enrolled in TR3 during the last three weeks. 

### 4.4. Interpretation of the Spectral Analysis Observations

Reduced access to feed as a possible interpretation of cow exposure to specific tie-rail configuration will probably affect the concentrations of several milk components simultaneously. The described methodology in this paper could detect a trend in changes of multiple milk components that could be attributed to the effect of changing tie-rail configuration before obtaining the concentrations of specific milk components. For example, this methodology concluded that lactose had an inversed relationship with fatty acids, BHB, acetone and citrate in milk samples collected during the last three weeks of the trial. The inverse relationship between lactose and BHB was confirmed by the concentrations of lactose and BHB reported by the milk analyzer (Table 5). During the last three weeks, the average lactose content was 4.62 and 4.60% for TR1 and TR3, respectively, and the average BHB content was 0.05 and 0.06% mmol/L for TR1 and TR3, respectively. For week 9, the average lactose content was 4.63 and 4.59% for TR1 and TR3, respectively. For week 10, the average BHB content was 0.05 and 0.07 mmol/L for TR1 and TR3, respectively. Increased levels of BHB, acetone, citrate and decreased levels of lactose are indicators of elevated body fat mobilization or negative energy balance [4,24], which suggests that cows assigned to TR3 might have been experiencing increased body fat mobilization in comparison to cows assigned to TR1 during the last three weeks of the trial [2]. This observation suggested that the TR3 configuration might have been obstructing cows’ access to feed. 

The interpretations of the spectra analysis regarding the effect of the tie-rail configuration on cow comfort was corroborated by observations of neck injuries collected during the animal trial. TR3 recorded increased injuries on two locations on the cow’s neck and those injuries might have been obstructing the cows from accessing feed [50]. While both TR1 and TR3 showed increased injuries on the proximal area of the cow’s neck (higher portion, closest to the body), TR3 was the only treatment out of the four tested treatments to additionally show increased injuries on the medial area of the cow’s neck (lower portion, closest to the head) [50]. These injuries resulted from the cows putting pressure on their neck through repeated contact with the tie-rail, while transitioning from lying to standing positions and possibly to reach feed [3]. The appearance of neck injuries at two locations of the neck indicates that TR3 tie-rail configuration was probably obstructing the cow access to feed, which may have resulted in a possible reduced feed intake (not measured in the trial) that led to an elevated body fat mobilization. In addition, no significant difference in eating–rumination time was found between the tie-rail treatments at any time point of the trial [62]. It must be noted that the effect was limited, and the milk composition dataset did not indicate any clinical issues with the cows assigned to TR3 or changes in energy reserves estimable using visual body condition scoring (BCS; no differences reported in St John et al., 2021 Supplementary Table S4 [62]). In cases of detectable clinical issues such as subclinical ketosis, BHB concentration increases to >0.1 mmol/L [63]. None of the reported averages of BHB exceeded this threshold in any treatment during this trial. Our results show that this spectral analysis approach has the potential to detect, through changes in milk composition, modifications of welfare status measurable, in the case of our study, with the appearance of body injuries, but also detected the trend in an early stage of body fat mobilization before it could be diagnosed.

To summarize, the described methodology in this paper combined tools used in FTIR spectroscopy and animal behavioral science, namely PCA and mixed modelling, to study the relationship between milk composition and dairy cow housing conditions providing a varying level of welfare in the controlled design trials. This methodology will open the door to study animal welfare from a novel angle, which will eventually help dairy herd improvement agencies provide new services for dairy farmers to improve animal welfare based on milk spectra that are routinely recorded, as an alternative to costly on-farm visits [1]. To our knowledge, this approach in studying animal welfare has never been reported before.

## 5. Conclusions

In this paper, a methodology of spectral analysis was developed to study the relationship between animal welfare and milk FTIR spectral data in the context of controlled-design animal trials by combining PCA with mixed modelling. Different tie-rail configurations for dairy cows housed in tie stalls were used as a proxy for changing animal welfare to test this methodology, which was successfully applied to the FTIR spectral data of milk samples collected during the trial. The proposed novel approach retained the multivariate structure of the FTIR spectral data and accommodated the use of the Mixed procedure as a powerful tool to test multiple experimental effects on milk FTIR spectral data. This methodology also revealed the tie-rail configuration treatment level that had a significant effect on milk spectral data with differences observed between the treatment with the most restricted movement and treatments with a less restricted movement. In addition, we could specify the spectral variables that captured differences in milk FTIR spectra attributed to the effect of the tie-rail configuration treatment level and milk components that might be assigned to these spectral variables. The described methodology of spectral analysis provides a new angle to study animal welfare in dairy cattle and enables field applications to help identify animal welfare issues using routinely collected milk spectra as an alternative to current assessment methods requiring an on-farm visit.

## Figures and Tables

**Table 1:** IR band centers of minor milk components detected in the FTIR spectra of spiked milk samples and aqueous solutions of the respective molecules.

Molecule	Band Centers in Milk cm^−1^ (Second Derivative)	Band Centers in Water cm^−1^ (Second Derivative)
Urea	1457, 1156	1461, 1160
β-Hydroxybutyric acid (BHB)	2926, 1554, 1405, 1316, 1077	2981, 1559, 1404, 1311, 1269, 1207, 1130, 1060, 948
Acetone	1690, 1414, 1373, 1239	1689, 1424, 1370, 1239, 1096
Citrate	2926, 1557, 1394, 1248, 1078	2923, 1581-1566, 1390, 1288, 1093
Acetate	1551, 1414	1554, 1416, 1348, 1060, 1021, 933
Phosphate	1156, 1077, 940	1261, 1236, 1160, 1077, 941
Ammonium chloride	1457	1454
Linoleic acid (fatty acid)	3012, 2927, 2857, 1705, 1581, 1554, 1458, 1408, 987	3011, 2929, 2861, 1597, 1554, 1458, 1405
Creatine	1538, 1396, 1311, 1106, 980	2950, 2835, 1538, 1431, 1396, 1307, 1168, 1107, 1049, 976
Histamine	3012, 2857, 1581, 1457, 1315, 1033, 987	3008, 2888, 1573, 1488, 1310, 1033, 987, 941
Orotic acid	1700, 1500, 1377, 1033	1700, 1497, 1377, 1014
Hippuric acid	1581, 1400, 1307	1584, 1489, 1396, 1301

**Table 2:** Principal components extracted from the raw, FD, vector normalized (VN) raw, VN-FD spectral datasets of the short-term and long-term milk spectral averages for the tie-rail trial. The table also lists *p* values obtained from the SAS mixed procedure for the treatment, start and block effects that are tested in this trial. Values in bold indicate principal components that revealed significant treatment effect.

**Long Term**							
Spectral Dataset	Meaningful PC	Eigenvalue	Explained Variance %	Cumulative Explained Variance %	*p* Values		
					Treatment	Start	Block
Raw	PC1	144.01	51.61	51.62	0.2897	0.0768	0.0081
	PC2	83.97	30.10	81.71	0.9753	0.2931	0.1052
	PC3	38.26	13.71	95.43	0.7750	0.0013	0.0001
	PC4	5.36	1.92	97.35	0.0836	0.7465	0.2169
	PC5	2.70	0.97	98.31	0.3495	0.1821	0.0859
FD	PC1	161.77	58.19	58.19	0.3120	0.1375	0.0091
	PC2	39.03	14.04	72.23	0.4568	0.0130	0.0010
	PC3	33.63	12.10	84.33	0.7935	0.0307	0.0388
	PC4	12.46	4.48	88.81	0.0519	0.0817	0.0071
	PC5	7.31	2.63	91.44	0.6068	0.0001	0.4963
	PC6	5.39	1.94	93.38	0.0371	0.0238	0.0464
	PC7	4.27	1.54	94.92	0.8602	0.7468	0.7219
VN Raw	PC1	189.23	68.07	68.07	0.4486	0.3247	0.0941
	PC2	62.68	22.55	90.62	0.9549	0.1570	0.0003
	PC3	11.23	4.04	94.66	0.6285	0.6290	0.1392
	PC4	6.49	2.34	96.99	0.0462	0.0695	0.0223
VN-FD	PC1	161.38	58.05	58.05	0.4429	0.2729	0.1311
	PC2	52.76	18.98	77.30	0.3412	0.0794	0.0001
	PC3	17.70	6.37	83.40	0.0698	0.2443	0.0145
	PC4	10.96	3.94	87.34	0.4485	0.0001	0.0513
	PC5	7.23	2.60	89.94	0.1883	0.0031	0.1370
	PC6	5.10	1.84	91.77	0.6687	0.2201	0.8147
	PC7	3.82	1.37	93.15	0.0106	0.5590	0.0600
	PC8	3.32	1.20	94.34	0.1827	0.1467	0.3407
	PC9	3.05	1.10	95.44	0.5853	0.9014	0.3648
Short Term							
Spectral Dataset	Meaningful PC	Eigenvalue	Explained Variance %	Cumulative Explained Variance %	*p* Values		
					Treatment	Start	Block
Raw	PC1	120.20	43.24	43.24	0.8027	0.2834	0.3742
	PC2	116.59	41.94	85.17	0.4505	<0.0001	0.0024
	PC3	28.43	10.23	95.40	0.9673	0.0022	0.0005
	PC4	4.70	1.69	97.09	0.7538	0.5885	0.2833
	PC5	3.42	1.23	98.32	0.4750	0.0667	0.1672
FD	PC1	143.72	51.70	51.70	0.7183	0.8642	0.2918
	PC2	53.42	19.22	70.916	0.8662	<0.0001	0.0028
	PC3	30.73	11.05	81.97	0.7711	0.0011	0.0509
	PC4	14.51	5.22	87.19	0.5312	0.0157	0.0699
	PC5	8.54	3.07	90.26	0.4634	0.0012	0.0001
	PC6	7.12	2.56	92.82	0.9830	0.4151	0.0185
	PC7	4.03	1.45	94.27	0.6678	0.1466	0.0445
	PC8	3.38	1.22	95.49	0.9792	0.2750	0.7527
VN Raw	PC1	184.33	66.305	66.305	0.8430	0.0835	0.4132
	PC2	59.13	21.27	87.57	0.8334	0.2707	0.0002
	PC3	18.65	6.70	94.28	0.3532	0.0001	0.0252
	PC4	6.05	2.18	96.46	0.3044	0.0524	0.2503
	PC5	3.00	1.08	97.54	0.5695	0.0047	0.1282
	PC6	2.14	0.77	98.31	0.3011	0.1517	0.0000
	PC7	1.42	0.51	98.82	0.2854	0.0331	0.2038
VN-FD	PC1	157.19	56.54	56.54	0.8203	0.1957	0.5475
	PC2	53.57	19.26	75.80	0.9078	0.4261	0.0057
	PC3	15.28	5.50	81.30	0.6044	0.0272	0.0968
	PC4	11.74	4.22	85.52	0.3441	<0.0001	0.0070
	PC5	7.86	2.83	88.35	0.3957	0.3243	<0.0001
	PC6	6.25	2.25	90.60	0.6979	0.0064	0.1319
	PC7	5.70	2.05	92.64	0.7479	0.9305	0.9583
	PC8	4.20	1.51	94.15	0.9162	0.5622	0.9332
	PC9	2.99	1.08	95.23	0.2639	0.8408	0.2663
	PC10	2.89	1.04	96.27	0.6651	0.2540	0.3726

**Table 3:** Least squares means produced by the mixed procedure for the scores of PC7 extracted from long-term VN-FD spectral average dataset and that revealed a significant treatment effect. This table shows that TR3 scores were significantly different from the other treatments (bolded). DF = degrees of freedom.

Treatment	Estimate	Standard Error	DF	t Value	*p* Value
**1**	0.6846	0.4959	30	1.38	0.1776
**2**	0.5001	0.5267	30	0.95	0.3499
**3**	−1.4596	0.4652	30	−3.14	**0.0038**
**4**	0.4188	0.4652	30	0.9	0.3752

**Table 4:** Differences of least squares means for PC7 scores. The Scheffé adjusted *p* values show that TR3 scores are significantly different from TR1 scores (bolded). DF = degrees of freedom.

Treatment	Treatment	Estimate	Standard Error	DF	t Value	*p* Value	Scheffé Adj. *p* Value
1	2	0.1845	0.7097	30	0.26	0.7967	0.9953
**1**	3	2.1442	0.68	30	3.15	0.0037	0.0332
1	4	0.2658	0.68	30	0.39	0.6986	0.9845
2	3	1.9597	0.7027	30	2.79	0.0091	0.0711
2	4	0.08133	0.7027	30	0.12	0.9086	0.9996
3	4	−1.8784	0.6579	30	−2.86	0.0077	0.0622

**Table 5:** Milk composition data ± SD for weeks 8–10 for the tie-rail trial with long-term averages by treatment.

Treatment	TR1 ^1^				TR2 ^1^			
Week	8	9	10	Avg.	8	9	10	Avg.
Fat %	4.22 ± 0.70	4.16 ± 0.71	4.10 ± 0.46	4.16 ± 0.62	4.20 ± 0.57	4.28 ± 0.63	4.16 ± 0.64	4.21 ± 0.59
Protein %	3.44 ± 0.32	3.44 ± 0.27	3.44 ± 0.26	3.44 ± 0.27	3.37 ± 0.33	3.38 ± 0.31	3.44 ± 0.31	3.39 ± 0.31
Lactose %	4.62 ± 0.12	4.63 ± 0.17	4.61 ± 0.18	4.62 ± 0.15	4.65 ± 0.16	4.65 ± 0.16	4.61 ± 0.19	4.64 ± 0.17
Urea mg/dL	14.29 ± 2.46	14.71 ± 2.90	13.90 ± 2.72	14.30 ± 2.63	14.03 ± 2.07	14.50 ± 3.50	13.54 ± 2.57	14.02 ± 2.71
BHB mmol/L	0.05 ± 0.03	0.05 ± 0.03	0.05 ± 0.03	0.05 ± 0.03	0.07 ± 0.03	0.06 ± 0.04	0.08 ± 0.03	0.07 ± 0.03
Treatment	TR3 ^1^				TR4 ^1^			
Week	8	9	10	Avg.	8	9	10	Avg.
Fat %	3.89 ± 0.45	3.94 ± 0.54	3.92 ± 0.49	3.91 ± 0.48	3.73 ± 0.64	3.90 ± 0.60	4.11 ± 0.56	3.91 ± 0.60
Protein %	3.34 ± 0.27	3.33 ± 0.29	3.36 ± 0.31	3.34 ± 0.28	3.41 ± 0.29	3.39 ± 0.30	3.42 ± 0.31	3.41 ± 0.29
Lactose %	4.63 ± 0.16	4.59 ± 0.20	4.59 ± 0.21	4.60 ± 0.18	4.61 ± 0.10	4.60 ± 0.11	4.59 ± 0.13	4.60 ± 0.11
Urea mg/dL	12.84 ± 2.77	13.05 ± 2.15	12.59 ± 2.13	12.83 ± 2.31	13.35 ± 1.74	14.48 ± 2.37	14.57 ± 2.34	14.13 ± 2.18
BHB mmol/L	0.06 ± 0.02	0.06 ± 0.02	0.07 ± 0.02	0.06 ± 0.02	0.06 ± 0.02	0.05 ± 0.03	0.07 ± 0.02	0.06 ± 0.03

^1^ TR1 (122 and 36 cm; height from the stall base and forward position from the manger well, respectively), TR2 (122 and 18 cm), TR3 (112 and 18 cm), and TR4 (112 and 36 cm).

## Data Availability

The dataset generated or analysed during the current study are available from the corresponding author.
